# Ceruloplasmin as Redox Marker Related to Heart Failure Severity

**DOI:** 10.3390/ijms221810074

**Published:** 2021-09-17

**Authors:** Elżbieta Lazar-Poloczek, Ewa Romuk, Piotr Rozentryt, Sylwia Duda, Mariusz Gąsior, Celina Wojciechowska

**Affiliations:** 1 Second Department of Cardiology, School of Medicine with the Division of Dentistry, Medical University of Silesia, M. C. Skłodowskiej 10 Street, 41-800 Zabrze, Poland; wojciechowskac@wp.pl; 2 Department of Biochemistry, School of Medicine with the Division of Dentistry, Medical University of Silesia, Jordana 19 Street, 41-808 Zabrze, Poland; eromuk@gmail.com; 3 Department of Toxicology and Health Protection, School of Public Health, Medical University of Silesia, 41-902 Bytom, Poland; prozentryt@sum.edu.pl (P.R.); sduda@sum.edu.pl (S.D.); 4 Third Department of Cardiology, SMDZ in Zabrze, School of Medicine with the Division of Dentistry, 41-800 Zabrze, Poland; mgasior@sum.edu.pl

**Keywords:** ceruloplasmin, heart failure, oxidative stress, hepatic enzymes

## Abstract

This study examined ceruloplasmin levels in patients with HFrEF, depending on cardiopulmonary exercise testing (CPET) parameters; a correlation was found between ceruloplasmin (CER) and iron and hepatic status, inflammatory and redox biomarkers. A group of 552 patients was divided according to Weber’s classification: there were 72 (13%) patients in class A (peak VO_2_ > 20 mL/kg/min), 116 (21%) patients in class B (peak VO_2_ 16–20 mL/kg/min), 276 (50%) patients in class C (peak VO_2_ 10–15.9 mL/kg/min) and 88 (16%) patients in class D (peak VO_2_ < 10 mL/kg/min). A higher concentration of CER was found in patients with peak VO_2_ < 16 mL/kg/min and VE/CO_2_ slope > 45 compared to patients with VE/CO_2_ slope < 45 (escectively CER 30.6 mg/dL and 27.5 mg/dL). A significantly positive correlation was found between ceruloplasmin and NYHA class, RV diameter, NT-proBNP, uric acid, total protein, fibrinogen and hepatic enzymes. CER was positively correlated with both total oxidant status (TOS), total antioxidant capacity (TAC) and malondialdehyde. A model constructed to predict CER concentration indicated that TOS, malondialdehyde and alkaline phosphatase were independent predictive variables (R^2^ 0.14, *p* < 0.001). CER as a continuous variable was an independent predictor of pVO_2_ ≤ 12 mL/kg/min after adjustment for sex, age and BMI. These results provide the basis of a new classification to encourage the determination of CER as a useful biomarker in HFrEF.

## 1. Introduction

Heart failure with a reduced ejection fraction (HFrEF) is a complex disease affecting many pathways in the human body. Increases in oxidation stress, low-grade inflammation, or iron deficiency or anemia have been documented [1,2,3]. Due to a reduction in cardiac output leading to disturbances between ventilation and perfusion, patients do not tolerate physical exertion. The exercise intolerance manifested by dyspnea, fatigue and weakness during physical activity are the most frequent symptoms in chronic heart failure. The increase in ventilation is a compensation mechanism directly related to the severity of the disease [4]. In clinical practice, the stage of impaired exercise tolerance may be described by using NYHA classifications, assessing the distance traveled in the 6-min walk test or using the many prognostic parameters of cardiopulmonary exercise testing (CPET) [5,6,7]. Weber et al. introduced a classification based on peak oxygen uptake (pVO_2_), which, together with four-stage ventilator classification determined from the relationship between minute ventilation and carbon dioxide production (VE/VCO_2_ slope), better defines the severity, prognosis and mortality in HF populations [8,9]. These classifications with reference cutoffs of the peak VO_2_ and VE/VCO_2_ slope are used in HFrEF not only for risk prediction, but also for extrapolating appropriate timelines for further treatments such heart transplantations [7]. Over the years, other authors have suggested new prognostic cutoffs of peak VO_2_ because of changes in HF treatments over the last 20 years. The current guidelines for heart transplantation recommend that a cutoff for pVO_2_ ≤14 mL/kg/min should be used to guide treatments in patients intolerant to β-blocker therapy. In the presence β-blocker treatment, the cutoff value of ≤12 mL/kg/min is more useful for risk stratification and should be used to guide listing [10]. Ceruloplasmin (CER) is an enzyme from the group of multicopper oxidases containing seven copper atoms per molecule, synthesized primarily in the hepatocytes. Multicopper oxidases are capable of oxidizing substrates through the transfer of four electrons to oxygen. Ceruloplasmin carries more than 95% of the total copper in healthy human plasma [11]. CER is involved in Fe metabolism by the oxidation of Fe^2+^ (ferrous iron) into Fe^3+^ (ferric iron)—ferroxidase activity. In fact, ceruloplasmin is the main contributor to ferroxidase I activity in human plasma [12,13]. Furthermore, the physiological functions of CER are also the transport and delivery of copper to tissues associated with transferrin, which can carry only iron in a ferric state [14]. In the cell-culture induction of CER expression, hypoxia (or iron deficiency) was exhibited [15]. Macrophages during hypoxia preferentially divert the copper into CER [16]. Ceruloplasmin can inhibit lipid peroxidation and the Fenton reaction, acting as a circulating scavenger of superoxide anion radicals and protecting cells and tissues against the detrimental effects of free radicals [17]. Replaced attributes make CER an effective antioxidant that prevents the oxidative damage of proteins and lipids [18]. However, it has been observed that CER exhibits both antioxidant (protective) and pro-oxidant (vasculopathic) properties [19]. It is quite likely that CER as a NO-oxidase may decrease NO levels in the heart, resulting in enhanced oxidative stress [20]. In addition, some data suggest that elevated levels of CER are associated with increased risks of developing heart failure [21]. CER is useful to predict acute heart failure in patients with myocardial infarction [22]. Ceruloplasmin is known as a minor acute phase reactant mediated by cytokines that increase during infection, as well as in low-grade inflammation [23]. Positive correlations between *C*-reactive protein and CER in ischemic and non-ischemic heart failure were identified; however, CER was associated with heart failure only in non-ischemic patients. CER as a useful prognostic biomarker in heart failure has been described previously [21,24]. The pathogenetic involvement of ceruloplasmin in heart failure research is not clear.

The aim of this study was to examine ceruloplasmin in patients with heart failure related to cardiopulmonary exercise testing and assess the connection of ceruloplasmin with iron and hepatic status, and inflammatory and redox biomarkers.

## 2. Results

The study group of 552 patients was divided according to Weber’s classification: there were 72 (13%) patients in class A (peak VO_2_ > 20 mL/kg/min), 116 (21%) patients in class B (peak VO_2_ 16–20 mL/kg/min), 276 (50%) patients in class C (peak VO_2_ 10–15.9 mL/kg/min) and 88 (16%) patients in class D (peak VO_2_ < 10 mL/kg/min). Pharmacological therapy regimens were comparable among the groups in terms of the use of angiotensin-converting enzyme inhibitors (ACE-Is) or angiotensin receptor blockers (ARBs), beta-blockers, mineralocorticoid receptor antagonists (MRAs) and statins. Across the whole group, 542 (98.2%) patients received beta-blockers, and 170 (30.8%) patients had a peak VO_2_ result of ≤12 mL/kg/min.

### 2.1. Demographic, Clinical and Laboratory Characteristics of the Patients Depending on the Weber Classification

The characteristics of the patients in the individual subgroups, depending on the Weber classification and taking into account significant differences, are presented in Table 1. The groups were significantly different in terms of the NT-proBNP concentration, hemoglobin, albumin fibrinogen, *C*-reactive protein and bilirubin. The activity of hepatic enzymes AST and ALT was comparable, but ALP and GGTP activity increased from group A to D. Total oxidative status and total antioxidant capacity were similar in all subgroups. The concentration of lipid peroxidation product (MDA) was higher in groups C and D than in groups A and B. An increase in the concentration of CER was observed from groups one to four, whereas the concentration of SH decreased simultaneously.

### 2.2. Demographic, Clinical and Laboratory Characteristics of Patients Depending on CER Quartiles

Additional characteristics are presented in Table 2: four subgroups were distinguished depending on the quartiles of CER concentration.

Among the four groups, the following parameters were comparable without any significant differences: sex, age, BMI, LV EDV and LVEF; complete blood count; and biochemical parameters, such as iron concentration, AST and ALT activity, fasting glucose levels and lipid profile. The occurrence of NYHA class IV was the highest in the fourth quartile. Peak VO_2_ in the 6 min WT decreased progressively along with CER quartiles. Moreover, the groups differed significantly in protein, albumin, fibrinogen, *C*-reactive protein, bilirubin and uric acid concentrations.

Weber classes: class A peak VO_2_ >20 mL/kg per minute, class B peak VO_2_ 16–20 mL/kg per minute, class C peak VO_2_ 10–16 mL/kg per minute, class D peak VO_2_ ≤ 10 mL/kg per minute; BMI, Body Mass Index; 6-min WT, 6 min walk test; NYHA, New York Heart Association functional class; LVEF, left ventricle ejection fraction; LVEDV, ventricular end-diastolic volume; RV diameter, right ventricular diameter; NT-proBNP, *N*-terminal pro-B-type natriuretic peptide; RBC, red blood cells; WBC, white blood cells; PLT, blood platelets; AST, aspartate aminotransferase; ALT, alanine aminotransferase; GGTP, gamma-glutamyl transpeptidase; ALP, alkaline phosphatase; SH, sulfhydryl group; MDA, malondialdehyde; TAC, total antioxidant capacity; TOS, total oxidant status; CER, ceruloplasmin; DCM, dilated cardiomyopathy; ICD, Implantable Cardioverter Defibrillator; NS, non-sinificant.

### 2.3. CER and NYNA Class and Cardiopulmonary Exercise Testing Results

**Figure 1:**
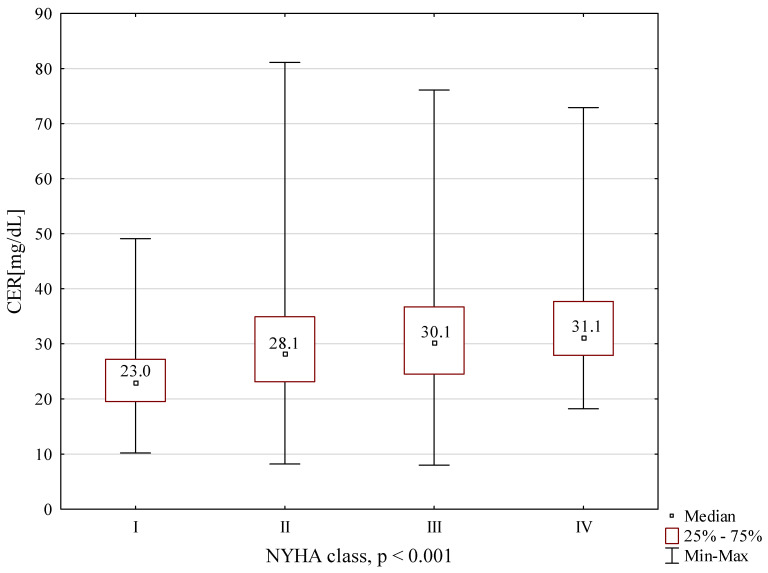
CER concentration in subgroups depending on NYHA classification.

**Figure 2:**
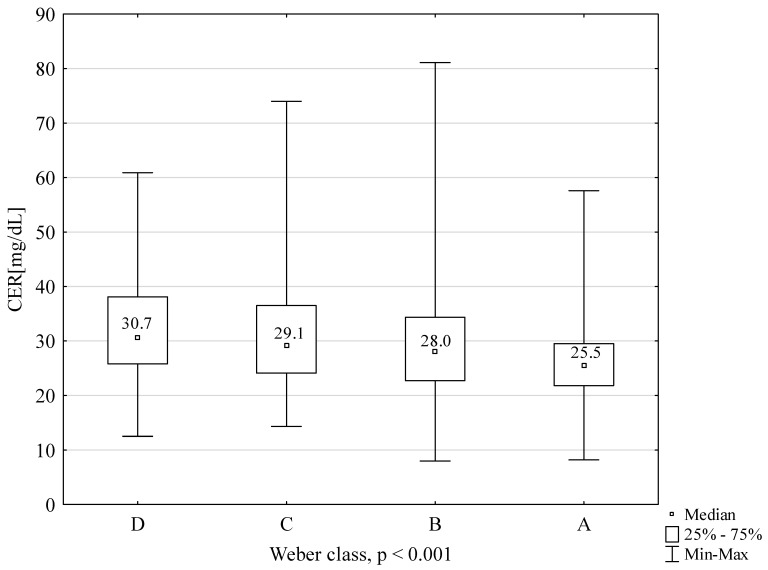
CER concentration in subgroups, depending on Weber classification.

**Figure 3:**
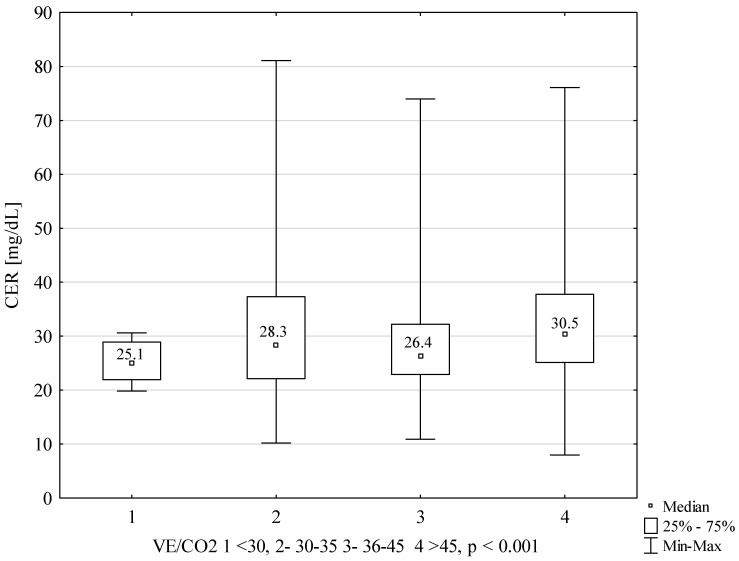
CER concentration in subgroups, depending on VE/VCO_2_ results.

**Figure 4:**
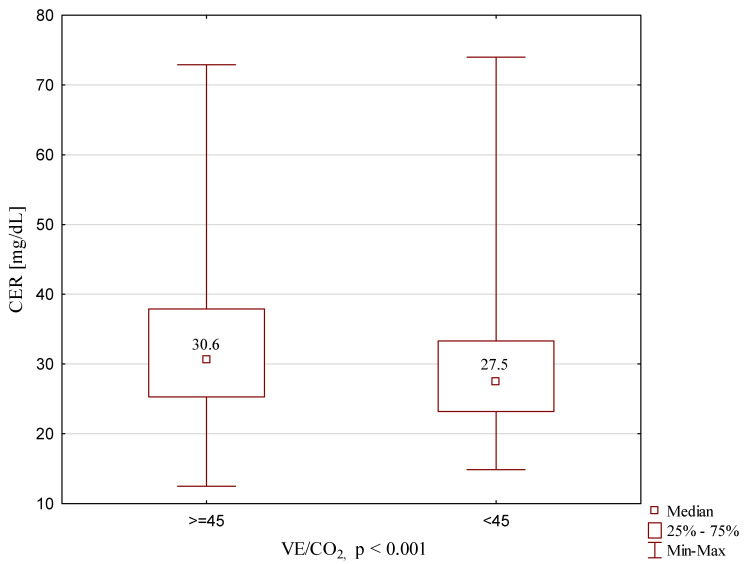
CER concentration in Weber class C and D, depending on VE/VCO2 results.

CER concentrations, depending on the NYHA classification, are presented in Figure 1. Significantly lower concentration values were found in NYHA 1 patients compared to each of the other groups. The following graphs present CER concentrations, depending on the Weber classification (Figure 2) and depending on VE/VCO2 (Figure 3). The concentrations of CER in patients in Weber classes C and D are also presented, taking into account the VE/CO_2_ result (cutoff 45) (Figure 4). A higher concentration of CER was found in patients with peak VO_2_ < 16 mL/kg/min and VE/CO_2_ slope > 45 compared to patients with VE/CO_2_ slope < 45 (rescectively CER 30.6 mg/dL and 27.5 mg/dL).

The logistic model for prediction the value of pVO_2_ ≤ 12 mL/kg/min revealed that CER as a continuous variable was an indicator of the poor test result. CER remains an independent predictor after adjustment for sex, age and BMI (Table 3).

### 2.4. The Association between CER Concentration and Demography, Clinical Parameters and Laboratory Parameters—Univariable Analysis

A significant positive correlation was found between CER and BMI (*r* = 0.09, *p* = 0.03), NYHA class (*r* = 0.19, *p* < 0.001), 6-min (*r* = −0.20, *p* < 0.001) WT LVEF (*r* = 0.10, *p* < 0.02) RV (*r* = 0.24, *p* < 0.001) diameter, NT-proBNP (*r* = 0.17, *p* < 0.001), uric acid (*r* = 0.15, *p* < 0.001), total protein (*r* = 0.20, *p* < 0.001), fibrinogen (*r* = 0.12, *p* = 0.004), bilirubin (*r* = 0.19, *p* < 0.001), ALP (*r* = 0.18, *p* < 0.001) and GGTP (*r* = 0.12, *p* = 0.004). CER correlated positively with both TOS (*r* = 0.25, *p* < 0.001) and TAC (*r* = 0.19, *p* < 0.001). Moreover, a positive correlation with MDA (*r* = 0.25, *p* < 0.001) and a negative with SH (*r* = −0.35, *p* < 0.001) were indicated.

### 2.5. The Association between CER Concentration Laboratory Parameters—Multiple Linear Regression

Significant variables correlated with CER included to a backward stepwise selection process. As a result, the model with three parameters was calculated. The model characteristics are presented in Table 4 and Table 5.

## 3. Discussion

Most data on the involvement of CER in cardiovascular diseases are associated with atherosclerosis and coronary artery disease [20,25]. However, the increased concentration of CER in heart failure was also indicated [24,26]. To the best of our knowledge, this is the first paper which has estimated CER levels in patients with HFrEF depending on symptom severity, assessed not only by subjective NYHA classification, but CPET results. In addition, the association of CER with known potentially pathological disorders, such as abnormal congestive hepatic status, iron deficiency, low-grade inflammation and oxidative stress, was estimated. Increases in CER concentration, depending on the extent of heart failure, were described by Yifei Xu et al. [27]. Researchers have indicated the correlation between NYHA classification only in patients with non-ischemic cardiomyopathy; this correlation was independent of other risk factors (gender, smoking, alcohol consumption, hypertension, diabetes mellitus, AST, uric acid, CKMB, CRP and LVEF). This association was not observed by Cabassia et al. [26], maybe because their study group included older-aged patients and a relatively small proportion of the patients had HFrEF (only 39%).

In our study, the highest serum CER levels were observed in patients with peak VO_2_ < 16 mL/kg/min (Weber classes C and D) and VE/VCO_2_ > 45. CER, after being adjusted for sex, age and BMI, remained an independent predictor of peak VO_2_ < 12 mL/kg/min. Previous studies have demonstrated that hypoxia increases the expression of the CER gene [15,28]. In all probability, this is the reason why we identified an association between the extent of HF characterized by peak VO_2_ in CPET in our cohort. Additionally, the highest serum CER levels were exhibited in patients with reduced peak VO_2_ and elevated VE/VCO_2_. An elevated VE/VCO_2_ response is associated with increased ventilation–perfusion mismatching (adequate ventilation and poor perfusion) [29,30].

This may also reflect a significant relationship with abnormally elevated chemoreceptor and ergoreceptor sensitivity, both of which contribute to an exaggerated ventilatory response to exercise. Our results showed that CER had a weak negative linear correlation with left-ventricular ejection fraction LVEF. Yifei Xu at al. [27], in contrast to Hammadach et al., noticed an equal association [21].

CER as a protein is synthesized by the liver. Our data suggest that serum CER levels are directly proportional to the total protein; however, they are not correlated with the albumin concentration or acute-phase reactants (such as CRP or fibrinogen). Similar to Yifei Xu et al. [31], we established a relationship between CER and CRP; nevertheless, further analysis showed that it was not the severity of inflammation, but the total oxidative activity that determined the higher CER concentration. Recent studies have indicated the impact of CER on ox-LDL. Our findings suggest that serum CER levels are correlated with the concentration of lipid peroxidation products, such as MDA. Moreover, this relationship has also been demonstrated in patients with arthritis [32].

Additionally, it is interesting to observe the significant negative linear correlation between CER and thiol groups (PSH). In contrast to our findings, Sarkar A. et al. showed that protein thiols were correlated positively with CER in type 2 DM patients compared with healthy controls [33]. Proteins with thiol groups are an important factor in balancing oxidative stress, due to their reducing properties. The oxidation of thiol groups is a reversible reaction; thus, produced disulfide links can be reduced again to thiol groups (dynamic thiol–disulfide homeostasis). There could be increases in oxidation processes (TOS) in HF and compensatory increases in antioxidant defense (TAC) and depletions of SH. TOS and MDA were identified as independent predictors of the CER concentration; therefore, their increases may be compensatory. However, due to the possible oxidation or nitrilation of amino acids, CER may lose its antioxidant character as ferroxidase [26].

In our study, positive associations between the CER concentration and certain protein enzymes (total alkaline phosphatase activity (ALP) and gamma-glutamyl transpeptidase (GGTP)) were detected. The enzyme alkaline phosphatase is important in serum analyses, and its elevation has been observed in the presence of bone and liver diseases [34]. High ALP and GGTP activities are known indicators of bile duct obstructions. CER is also correlated positively with elevated bilirubin, although decreased serum CER has been demonstrated in patients with hepatic fibrosis and chronic hepatitis [31]. Multiple regression analysis showed that ALP activity is an independent predictor of increased CER serum levels. A challenging issue that arises in this field is the compensatory increase in CER level as a protective and antioxidant role, even though elevated CER appears to be connected with increased HF mortality [21,24]. In the last decade, other investigators have provided evidence of the role of CER in the conversion of Fe^2+^ to Fe^3+^, which prevents the Fenton reaction [11,35]. However, some studies have indicated that CER, as well as vitamin C and E, exhibits not only antioxidant but also pro-oxidant properties, depending on the environment. It is important to emphasize that, despite the participation of copper in iron metabolism, our results did not show any correlation between CER and iron or hemoglobin concentrations. A recent study described low serum iron as an adverse prognostic factor in heart failure [36], which did not correlate with CER in our patient population.

## 4. Materials and Methods

### 4.1. Study Population and Clinical Assessment

We analyzed the data of 741 patients with symptomatic heart failure with reduced ejection fraction (HFrEF) that were included in a previous study [24].

Briefly, those in this cohort with HFrEF were referred to our inpatient clinic as potential candidates for heart transplantation. The main inclusion criteria were reduced left ventricular ejection fraction (LVEF ≤ 40%) and stable symptomatic heart failure, despite having received optimal pharmacological therapy according to the current ESC guidelines for the diagnosis and treatment of acute and chronic heart failure 2008–2016 [37,38,39] for at least 3 months. We excluded 62 patients with diagnosed chronic obstructive pulmonary disease from the evaluation, as well as 131 current cigarette smokers and 25 patients with musculoskeletal dysfunction syndrome, due to the potential confounding impacts on the results of cardiopulmonary exercise testing. Ultimately, the results of 552 patients were analyzed. All patients underwent a clinical assessment of disease severity: transthoracic echocardiography (TTE) evaluation, end-diastolic volume (EDV) and end-systolic volume (ESV) in the biplane TTE were measured by Simpson’s method of discs. The ejection fraction was calculated by using the following equation:

EF = (EDV − ESV)100% ÷ EDV. (1)

Both NYHA class and cardiopulmonary exercise testing (CPET) were used to estimate exercise tolerance. CPET was performed by using a VMAX—oxygen consumption scanner (General Electric, Milwaukee, WI, USA). The patients underwent a symptom-limited treadmill exercise test (modified Bruce’s protocol) after a 5-min rest period. Respiratory gas-exchange data, minute ventilation and oxygen consumption were collected continuously. Peak oxygen consumption (pVO_2_) was measured as an arithmetic mean of values recorded within the last 30 s before the cessation of exercise and was expressed in mL/kg/min. All the procedures were carried out in accordance with the 1975 Declaration of Helsinki and its revision in 2008. All the participants provided written informed consent prior to enrollment in the study. The local ethics committee of Silesian Medical University approved the study protocol (NN-6501-12/I/04).

### 4.2. Biochemical Methods

Patients’ blood samples obtained at the study inclusion were separated by centrifugation at 1500× *g* for 10 min (MPW, Warsaw, Poland) and partially stored at −70 °C until being assayed. Serum protein, albumin, fibrinogen, CRP, alanine aminotransferase, aspartate aminotransferase, gamma-glutamyl transferase (GGTP), alkaline phosphatase, bilirubin, lipid parameters, serum iron, creatinine, glucose and uric acid concentrations were measured by colorimetric methods (Cobas 6000 e501; Roche, Basel, Switzerland). Hemoglobin, leukocytes and platelets were measured with the use of a MEDONIC M32C analyzer (Alpha Diagnostics, Warsaw, Poland). NT-proBNP was measured with the use of the chemiluminescence method (Cobas 6000 e501).

Serum CER concentration was determined according to the spectrophotometric Richterich method [40]. Ceruloplasmin catalyzes the oxidation of colorless p-phenylenediamine to blue-violet dye. The test sample contained twenty microliters of serum, whereas the control sample contained 20 μL of serum; 200 μL of sodium azide solution was added to stop the reaction. In the next step, 1 mL of p-phenylenediamine dihydrochloride in acetate buffer was added to both samples. After a 15 min incubation, 200 μL of sodium azide was added to the test sample. Finally, after a 15 min incubation, the absorbance of test and control samples was measured at 560 nm, using a PerkinElmer VICTOR-X3 plate reader (Waltham, MA, USA). The intra-assay coefficient of variation was 3.7%, and the intra-assay precision was 4%.

Spectrophotometric Erel’s method was used to determine total oxidant status (TOS). In this method, we have measured the color intensity of complex of Fe^3+^ ions and Xylenol orange in an acidic environment. TOS is expressed in mmol/L [41].

TAC was measured by colorimetric Erel’s methods, based on 2,2′-azino-bis(3-ethylbenzothiazoline-6-sulfonate) (ABTS+) reaction (Sigma-Aldrich, Saint Louis, Missouri, USA). In this method a colorless reduced ABTS molecule, is oxidized to blue-green ABTS⋅+. After mixing the colored ABTS⋅+ with any substance that can be oxidized, it is reduced to its original colorless reduced form. Reacted substance is oxidized. TAC is expressed in mmol/L [42].

The Koster method, using 5,5′-dithiobis (2-nitrobenzoic acid) or DTNB (Sigma-Aldrich, Saint Louis, MO, USA), was used to measure the concentration of sulfhydryl groups (PSH) in serum. After reduction by the sulfhydryl-group-containing compounds, DTNB produced the yellow-colored anionic 5-thio-2-nitrobenzoic acid. The absorbance was measured at a wavelength of 412 nm with a Shimadzu 1700 UV–VIS spectrophotometer (Mettler Toledo, Columbus, OH, USA). PSH concentration was expressed in μmol/g protein [43]. Malondialdehyde (MDA) was measured by Ohkawa’s method. In this method, the reaction of lipid peroxides with thiobarbituric acid with spectrofluorimetric detection was used. The excitation wavelength was 515 nm, and the emission wavelength was 552 nm. The MDA concentration was expressed in μmol/L and calculated from the standard curve prepared for 1,1,3,3-tetraethoxypropane [44].

### 4.3. Statistical Analysis

Categorical data are displayed as proportions and were compared by using the chi-squared test with the Yates correction. The distributions of all the continuous variables were evaluated with the Shapiro–Wilk test. Due to the abnormal distribution of most continuous variables, the continuous data are presented as medians with the first and third quartiles. Study participants were divided into subgroups based on CPET results according to their Weber class (A–D). The Shapiro–Wilk test was used to evaluate the distribution of all continuous variables. Continuous data are presented as the median with the first and third quartiles (because of non-normal distribution for more parameters). Categorical data are presented as absolute numbers and percentages. Kruskal–Wallis ANOVA tests were performed to compare continuous data. The prevalence of comorbidities was compared by using the chi-squared test with the Yates correction. Pearson correlation coefficients were calculated to describe univariable associations between ceruloplasmin concentration and demography; clinical parameters; laboratory evaluations, including acute phase protein and hepatic enzymes; and reduction–oxidation status. Multiple linear regression analysis was used to determine laboratory predictors of CER as a dependent variable, incorporating parameters with univariable associations significant at the *p* < 0.1 level. Collinearity between independent variables was assessed. There was significant collinearity between hepatic, redox and inflammatory parameters; therefore, we examined their independent association with CER by separate models. The model was constructed on the basis of the backward stepwise method. To assess the independent contribution of CER, sex, age and BMI on the pVO_2_ value ≤ 12 mL/min/kg, logistic regression was used. Statistical analyses were performed by using STATISTICA 12.0, (StatSoft Inc, Tulsa, OK, USA) albo STATISTICA 13.1 PL software (StatSoft, Cracow, Poland), assuming a level of *p* < 0.05 as statistically significant. All the subjects who participated in the study provided informed consent to allow the analysis of data for research purposes, and all the subjects gave their agreement in written form. The study was approved by the local Ethical Review Board (according to the study protocol of Silesian Medical University, KNW/0022/KB1/9/13).

## 5. Conclusions

These results complement the current knowledge supporting the use of CER as a significant biomarker in heart failure with reduced ejection fraction (HFrEF). To the best of our knowledge, this is the first study to have evaluated CER in patients with HFrEF depending on CPET results. CPET more precisely reflects the severity of disease than subjective NYHA classifications; additionally, in the heart-failure population, mixed Weber and ventilatory classifications maintained their prognostic properties. Over time, new cutoffs have been proposed because of impacts of therapies, such as beta-blockers or other interventions. CER adjusted for sex, age and BMI was an independent predictor of peak VO_2_ ≤ 12 mL/kg/min. Patients with reduced peak VO_2_ (Weber class C and D) and elevated VE/VCO_2_ demonstrated the highest serum CER levels. The VE/VCO2 slope predicts not only reduced peak cardiac output, but also represents pulmonary circulation dysfunction. These results may encourage the utilization of CER as a biomarker in HFrEF.

Furthermore, our study demonstrated positive associations between CER, produced in the liver, and the hepatic enzymes GGTP and ALP; however, we did not observe correlations between CER and iron or hemoglobin concentrations, despite the contribution of copper to iron metabolism. Moreover, we assessed the correlations between CER and inflammatory and redox biomarkers, which allowed us to conclude that CER is more closely related to oxidative stress than inflammation in heart failure. We believe that further studies should be performed to determine new roles of CER multifunctionality.

## 6. Study Limitation

There was a lack of determination of free copper. Moreover, only CER concentrations were determined; there was no enzymatic activity assessment. Finally, this study employed one-point testing.

## Figures and Tables

**Table 1:** The characteristics of the patients in the individual subgroups depending on the Weber classification.

	Class A *n* = 72	Class B *n* = 116	Class C *n* = 276	Class D *n* = 88	*p*
Female *n* (%)	7 [9.72]	9 [7.76]	50 [18.1]	15 [17.0]	<0.05
Age [years]	50.0 [40.0–58.0]	53.0 [48.0–58.0]	55.0 [51.0–59.0]	56.0 [50.0–61.0]	<0.001
BMI [kg/m^2^]	26.5 [23.7–29.2]	26.1 [23.7–28.5]	26.5 [23.6–29.3]	26.3 [22.8–30.2]	NS
Exercise capacity, echocardiography					
NYHA class I/II/III/IV *n* [%]	20/39/13/0 [27.8/54.2/18.0/0]	10/58/46/2 [8.6/50.0/39.7/1.7]	2/89/155/30 [0.7/32.2/56.2/10.9]	0/10/54/24 [0/11.4/61.4/27.3]	<0.01
6-min WT [m]	455.5 [430.5–544.0]	400.0 [380.0–440.0]	347.0 [303.0–390.0]	267.0 [220.0–325.0]	<0.001
LVEF [mm]	25.5 [21.5–35.0]	25.0 [21.0–31.0]	23.0 [20.0–29.0]	22.0 [19.0–26.0]	<0.001
LVEDV [mL]	69.0 [63.0–75.0]	69.0 [63.0–76.0]	69.0 [64.0–76.0]	73.0 [66.0–77.0]	NS
RV diameter [mm]	29.0 [25.0–33.0]	28.0 [25.0–32.0]	30.0 [26.0–34.0]	31.0 [28.0–35.0]	<0.001
Laboratory parameters					
NT-proBNP [pg/mL] /100	657.8 [252.4–1378.5]	978.9 [505.7–1620.0]	1867.0 [762.5–3474.0]	2253.0 [1103.0–4399.0]	<0.001
RBC [1012 /L]	4.7 [4.4–5.0]	4.6 [4.3–5.0]	4.5 [4.2–4.9]	4.6 [4.2–4.9]	NS
WBC [109 /L]	6.5 [5.3–7.7]	7.0 [5.9–8.3]	7.0 [5.9–8.3]	6.4 [5.4–7.7]	NS
PLT [109 /L]	183.0 [152.0–223.5]	181.5 [139.0–217.0]	184.0 [154.0–219.0]	180.0 [145.0–236.0]	NS
Hemoglobin [mmol/L]	14,5 [13.9–15.2]	14.2 [13.4–15.0]	14.0 [13.1–15.0]	13.9 [12.7–14.8]	<0.01
Iron [µmol/L]	18.4 [15.1–23.2]	17.5 [13.3–22.2]	17.2 [12.0–22.6]	16.7 [11.8–19.6]	NS
Uric acid [µmol/L]/ 10	429.5 [354.0–487.5]	392.0 [322.0–480.0]	408.0 [323.0–516.0]	425.0 [351.0–559.0]	<0.05
Serum protein [g/L]	71.5 [67.0–76.0]	71.0 [68.0–74.0]	71.0 [67.0–75.0]	71.0 [66.0–76.0]	NS
Albumin [g/L]	43.0 [40.0–45.0]	42.5 [40.0–45.0]	42.0 [40.0–44.0]	41.0 [38.0–43.0]	<0.01
Fibrinogen [mg/dL]	349.5 [307.5–405.0]	366.0 [315.0–435.0]	402.0 [346.0–465.0]	431.0 [371.0–471.0]	<0.001
*C*-reactive protein [mg/L]	1.5 [0.8–2.9]	1.6 [0.9–4.2]	3.3 [1.6–7.0]	3.9 [2.3–7.5]	<0.001
Bilirubin [µmol/L]	11.3 [7.7–16.5]	11.4 [9.1–16.6]	15.2 [10.6–21.9]	17.3 [12.3–26.2]	<0.001
AST [IU/l]	24.0 [21.0–33.5]	23.0 [19.0–31.0]	23.0 [18.0–30.0]	23.0 [18.0–29.0]	NS
ALT [IU/l]	27.5 [19.5–42.0]	24.0 [19.0–34.0]	25.0 [17.0–36.0]	22.0 [15.0–31.0]	<0.05
GGTP [IU/l]	37.0 [20.0–84.5]	42.5 [25.0–100.0]	54.0 [29.0–107.0]	62.0 [33.0–117.0]	<0.01
ALP [IU/l]	63.0 [51.0–76.0]	60.5 [49.0–81.0]	70.0 [57.0–90.0]	81.0 [60.0–104.0]	<0.001
Fasting glucose [mmol/L]	5.4 [4.9–6.4]	5.6 [5.0–6.3]	5.6 [5.0–6.4]	5.4 [5.0–6.1]	NS
Total Cholesterol [mmol/L]	4.1 [3.6–5.4]	4.4 [3.7–5.2]	4.2 [3.6–5.0]	4.1 [3.3–5.2]	NS
Cholesterol HDL [mmol/L]	1.2 [1.0–1.5]	1.2 [0.9–1.6]	1.1 [0.9–1.4]	1.1 [0.9–1.4]	NS
Triglycerides [mmol/L]	1.4 [0.9–2.0]	1.2 [0.9–1.8]	1.2 [0.9–1.7]	1.1 [0.8–1.5]	NS
SH [µmol/g protein]	317.0 [235.2–368.0]	296.7 [229.5–360.0]	283.6 [212.4–347.9]	260.6 [225.1–318.8]	<0.05
MDA [µmol/L]	1.6 [1.3–2.2]	1.6 [1.3–2.0]	1.8 [1.4–2.1]	1.8 [1.55–2.15]	<0.05
TAC [mmol/L]	1.11 [0.99–1.22]	1.09 [0.99–1.22]	1.13 [1.02–1.25]	1.14 [1.06–1.27]	NS
TOS [mmol/L]	5.2 [4.2–6.5]	4.8 [4.2–6.0]	4.8 [4.1–6.1]	4.8 [4.1–6.0]	NS
CER [mg/dL]	25.5 [21.8–29.5]	2.0 [22.7–34.35]	29.1 [24.1–36.5]	30.7 [25.8–38.1]	<0.001
Comorbidities					
Ischemic DCM *n* [%]	54 [75.0]	102 [88.0]	244 [88.4]	79 [89.7]	<0.05
Diabetes *n* [%]	21 [29.1]	36 [31.0]	85 [30.7]	28 [31.8]	NS
Arterial hypertension *n* [%]	39 [54.2]	63 [54.3]	168 [60.9]	55 [62.5]	NS
Permanent atrial fibrillation; *n* [%]	8 [11.1]	32 [27.6]	73 [26.4]	34 [38.6]	<0.001
ICD presence *n* [%]	9 [12.5]	20 [17.2]	56 [20.2]	18 [20.4]	NS

Weber classes: class A peak VO_2_ >20 mL/kg per minute, class B peak VO_2_ 16–20 mL/kg per minute, class C peak VO_2_ 10–16 mL/kg per minute, class D peak VO_2_ ≤10 mL/kg per minute; BMI, Body Mass Index; 6-min WT, 6 min walk test; NYHA, New York Heart Association functional class; LVEF, left ventricle ejection fraction; LVEDV, ventricular end-diastolic volume; RV diameter, right ventricular diameter; NT-proBNP, *N*-terminal pro-B-type natriuretic peptide; RBC, red blood cells; WBC, white blood cells; PLT, blood platelets; AST, aspartate aminotransferase; ALT, alanine aminotransferase; GGTP, gamma-glutamyl transpeptidase; ALP, alkaline phosphatase; SH, sulfhydryl group; MDA, malondialdehyde; TAC, total antioxidant capacity; TOS, total oxidant status; CER, ceruloplasmin; DCM, dilated cardiomyopathy; ICD, Implantable Cardioverter Defibrillator; NS, non-sinificant.

**Table 2:** The characteristics of the patients in the individual subgroups, depending on the CER-concentration quartiles.

CER Quartiles [mg/dL]					
	1st <23.7 *n* = 139	2nd 23.7–28.70 *n* = 139	3rd 23.7–28.70 *n* = 137	4th >36 *n* = 137	*p*
Female n [%]	15 [10.8]	17 [12.2]	20 [14.6]	27 [19.7]	NS
Age [years]	54.0 [49.0–58.0]	54.0 [49.0–60.0]	54.0 [48.0–58.0]	55.0 [49.0–60.0]	NS
BMI [kg/m^2^]	26.7 [24.3–29.4]	27.0 [24.2–30.0]	26.4 [23.5–29.7]	26.0 [22.6–28.5]	NS
Exercise Capacity, Echocardiography					
NYHA n I/II/III/IV [%]	20/56/57/6 14.4/40.3/41.0/4.3	8/58/62/11 5.8/41.7/44.6/7.9	3/40/76/18 2.2/29.2/55.5/13.1	1/42/73/21 0.7/30.7/53.3/15.7	<0.001
Peak VO_2_ [ml/kg/min]	15.2 [12.2–19.2]	14.6 [12.0–17.5]	13.9 [11.2–17.0]	13.2 [10.6–16.4]	<0.001
VE/CO_2_ Slope	42.0 [37.0–50.0]	44.0 [38.5–51.0]	49.0 [41.0–57.0]	48.0 [41.0–57.0]	<0.001
6-min WT [m]	390.0 [335.0–440.0]	390.0 [343.0–432.5]	372.5 [284.0–423.0]	346.0 [267.0–390.0]	<0.05
LVEF [%]	25.0 [20.0–33.0]	24.0 [20.0–32.0]	24.0 [20.0–28.0]	22.5 [20.0–28.0]	NS
LVEDV [mL]	208.0 [163.0–264.0]	220.0 [163.0–287.0]	228.0 [190.0–286.0]	237.0 [168.0–293.0]	NS
RV diameter [mm]	27.0 [24.0–31.0]	29.0 [26.0–33.0]	30.0 [27.0–34.0]	31.0 [28.0–35.0]	<0.001
Laboratory Parameters					
NT-proBNP [pg/mL]	1038.0 [516.5–2141.0]	1369.5 [656.0–3476.0]	1603.0 [707.9–3259.0]	1701.5 [883.9–3642.0]	NS
RBC [10^12^/L]	4.5 [4.1–4.8]	4.6 [4.3–5.0]	4.6 [4.3–5.0]	4.6 [4.2–5.0]	NS
WBC [10^9^/L]	6.8 [5.5–8.2]	6.7 [5.4–8.1]	7.2 [5.8–8.6]	6.7 [6.1–7.9]	NS
PLT [10^9^/L]	180.5 [148.0–220.0]	182.5 [155.0–215.0]	190.0 [153.0–235.0]	170.5 [147.5–216.0]	NS
Hemoglobin [mmol/L]	13.9 [13.1–14.8]	14.0 [12.9–15.0]	14.3 [13.2–15.1]	14.0 [13.1–15.1]	NS
Iron [μmol/L]	16.8 [12.9–20.1]	17.7 [11.5–22.1]	17.2 [12.3–22.9]	17.3 [12.4–24.1]	NS
Uric Acid [μmol/L]/10	373.5 [331.0–446.0]	413.0 [328.0–500.0]	414.0 [316.0–507.0]	438.5 [327.5–557.5]	<0.05
Serum Protein [g/L]	70.0 [67.0–74.0]	69.0 [66.0–73.0]	73.0 [69.0–76.0]	73.0 [69.0–77.0]	<0.001
Albumin [g/L]	42.0 [40.0–44.0]	41.0 [39.0–43.0]	41.0 [39.0–44.0]	43.0 [40.0–45.0]	<0.01
Fibrinogen [mg/dl]	367.5 [322.0–438.0]	386.5 [330.0–441.0]	424.0 [359.0–481.0]	408.0 [341.0–489.0]	<0.001
*C*-reactive Protein [mg/L]	2.0 [0.9–4.9]	2.0 [1.1–5.9]	3.7 [1.9–7.2]	3.4 [1.7–7.5]	<0.001
Bilirubin [μmol/L]	11.6 [8.7–16.2]	13.8 [9.6–18.4]	15.1 [9.8–21.7]	16.8 [11.2–27.1]	<0.001
AST [IU/L]	22.5 [18.0–28.0]	23.0 [18.0–29.0]	25.0 [19.0–31.0]	24.0 [20.0–33.0]	NS
ALT [IU/L]	22.5 [17.0–34.0]	24.0 [17.0–34.0]	26.0 [18.0–35.0]	25.0 [18.0–38.0]	NS
GGTP [IU/L]	39.5 [23.0–74.0]	42.0 [25.0–87.0]	57.0 [29.0–119.0]	65.0 [30.5–152.0]	<0.001
ALP [IU/L]	65.0 [51.0–79.8]	63.0 [52.0–77.0]	74.0 [60.0–95.3]	75.5 [59.0–107.0]	<0.001
Fasting Glucose [mmol/L]	5.5 [5.0–6.3]	5.5 [4.9–6.2]	5.6 [5.0–6.8]	5.5 [5.0–6.2]	NS
Total Cholesterol [mmol/L]	4.2 [3.6–5.0]	4.2 [3.6–5.0]	4.2 [3.5–5.3]	4.5 [3.7–5.2]	NS
Cholesterol HDL [mmol/L]	1.2 [1.0–1.5]	1.1 [0.9–1.4]	1.1 [0.9–1.3]	1.1 [0.9–1.4]	NS
Triglycerides [mmol/L]	1.1 [0.8–1.6]	1.2 [0.9–1.7]	1.3 [1.0–1.7]	1.2 [0.9–1.7]	NS
SH [μmol/g protein]	304.4 [242.6–364.6]	307.4 [231.6–357.7]	290.9 [225.7–356.0]	234.8 [174.9–305.3]	<0.001
MDA [μmol/L]	1.7 [1.3–2.0]	1.6 [1.3–1.9]	1.7 [1.4–2.2]	2.0 [1.5–2.4]	<0.001
TAC [mmol/L]	1.1 [1.0–1.2]	1.1 [1.0–1.2]	1.1 [1.0–1.2]	1.2 [1.1–1.3]	<0.001
TOS [mmol/L]	4.3 [3.3–5.1]	5.0 [4.3–5.9]	5.4 [4.4–6.5]	5.2 [4.3–6.8]	<0.001
Comorbidities					
Ischemic DCM *n* [%]	124 [89.2]	113 [81.3]	118 [84.9]	124 [89.2]	NS
Diabetes *n* [%]	36 [25.9]	43 [30.9]	48 [34.5]	43 [30.9]	NS
Arterial Hypertension *n* [%]	80 [57.6]	85 [61.2]	70 [50.4]	90 [64.7]	NS
Permanent Atrial Fibrillation; *n* [%]	20 [14.4]	37 [26.6]	41 [29.5]	49 [35.3]	<0.001
ICD Presence *n* [%]	17 [12.2]	24 [17.3]	36 [25.9]	26 [18.7]	NS

BMI, Body Mass Index; 6-min WT, 6 min walk test; NYHA, New York Heart Association functional class; peak VO_2_, peak oxygen uptake; VE/VCO_2_ slope, ventilation/carbon dioxide production; LVEF, left ventricle ejection fraction; LVEDV, ventricular end-diastolic volume; RV diameter, right ventricular diameter; NT-proBNP, *N*-terminal pro-B-type natriuretic peptide; RBC, red blood cells; WBC, white blood cells; PLT, blood platelets; AST, aspartate aminotransferase; ALT, alanine aminotransferase; GGTP, gamma-glutamyl transpeptidase; ALP, alkaline phosphatase; SH, sulfhydryl group; MDA, malondialdehyde; TAC, total antioxidant capacity; TOS, total oxidant status; DCM, dilated cardiomyopathy; ICD, Implantable Cardioverter Defibrillator; NS, non-significant.

**Table 3:** Logistic regression analysis of CER, sex, age and BMI for pVO_2_ value differentiation.

Explanatory Variables	pVO_2_ ≤ 12 mL/kg/min					
	OR	95% CI	*p*	Adjusted OR	95% CI	*p*
Male	1			1		
Female	2.396	1.537–3.737	<0.001	2.260	1.418–3.602	<0.001
Age	1.028	1.011–1.046	0.001	1.031	1.013–1.049	<0.001
BMI	1.029	0.992–1.067	0.130			
CER	1.024	1.008–1.040	0.003	1.022	1.006–1.039	<0.05

BMI, Body Mass Index; CER, Ceruloplasmin.

**Table 4:** Backward stepwise multiple linear regression analysis of predictors of CER concentration.

Dependent Variables	*b* *	Standard Error	β	T	*p*
Intercept Term			14.886	1.830	<0.001
TOS	0.235	0.040	0.997	0.171	<0.001
MDA	0.194	0.040	3.776	0.785	<0.001
ALP	0.178	0.040	0.045	0.010	<0.001

*b* *, unstandardized regression coefficient; β, regression standardized coefficient; TOS, total oxidant status; MDA, malondialdehyde; ALP, alkaline phosphatase.

**Table 5:** Model characteristics.

R	0.38
R^2^ Value	0.14
The Adjusted R^2^ Value	0.13
*p*-Value	<0.001
Standard Error of the Estimate	9.61

The model was characterized the ability to predict the concentration of CER. A positive relationship was found between all its variables (TOS, MDA and ALP) and the concentration of CER.

## Data Availability

The original data is available after contact with the corresponding author.
